# In Silico Investigation into Cellular Mechanisms of Cardiac Alternans in Myocardial Ischemia

**DOI:** 10.1155/2016/4310634

**Published:** 2016-12-13

**Authors:** Jiaqi Liu, Yinglan Gong, Ling Xia, Xiaopeng Zhao

**Affiliations:** ^1^Department of Biomedical Engineering, Zhejiang University, Hangzhou 310027, China; ^2^Department of Mechanical, Aerospace, and Biomedical Engineering, University of Tennessee, Knoxville, TN 37996, USA

## Abstract

Myocardial ischemia is associated with pathophysiological conditions such as hyperkalemia, acidosis, and hypoxia. These physiological disorders may lead to changes on the functions of ionic channels, which in turn form the basis for cardiac alternans. In this paper, we investigated the roles of hyperkalemia and calcium handling components played in the genesis of alternans in ischemia at the cellular level by using computational simulations. The results show that hyperkalemic reduced cell excitability and delayed recovery from inactivation of depolarization currents. The inactivation time constant *τ*_*f*_ of L-type calcium current (*I*_CaL_) increased obviously in hyperkalemia. One cycle length was not enough for *I*_CaL_ to recover completely. Alternans developed as a result of *I*_CaL_ responding to stimulation every other beat. Sarcoplasmic reticulum calcium-ATPase (SERCA2a) function decreased in ischemia. This change resulted in intracellular Ca (Ca_*i*_) alternans of small magnitude. A strong Na^+^-Ca^2+^ exchange current (*I*_NCX_) increased the magnitude of Ca_*i*_ alternans, leading to APD alternans through excitation-contraction coupling. Some alternated repolarization currents contributed to this repolarization alternans.

## 1. Introduction

The mechanisms underlying ventricular arrhythmias are complex [1]. Ischemia is one of the main causes. Cardiac arrhythmias are produced by electrophysiological disturbances of the heart [1]. Three major pathophysiological conditions linked to acute myocardial ischemia have been identified, including elevated extracellular potassium, acidosis, and anoxia [2]. These conditions cause changes of electrical activities that produce the potent arrhythmia substrate.

T-wave alternans (TWA) can be used for predicting arrhythmogenesis in clinical practice [3]. TWA refers to beat-to-beat alternation in the morphology and amplitude of the ST-segment or T-wave magnitude [3]. Electrical instabilities in ischemia promote the occurrence of TWA. Animal experiments show that ischemia increases the magnitude of TWA [3]. Moreover, TWA alone can be identified as a strong indicator for ischemic cardiomyopathy [4]. It originates from action potential duration (APD) alternans at the cellular level [3].

To understand the mechanism of TWA, the study of APD alternans is necessary. APD alternans can be caused either by voltage instabilities (voltage-driven alternans) or by Ca^2+^ handling dynamics instabilities (Ca^2+^-driven alternans) or their interactions [5]. Because of the bidirectional coupling between membrane voltage kinetics and Ca handling dynamics, it is difficult to identify the exact mechanism of APD alternans [6, 7]. Voltage instabilities or Ca^2+^ handling instabilities affect alternans occurring through changes of ionic currents. Thus, there must exist ionic basis in the genesis of alternans. In order to explore the role of ionic currents in the genesis of alternans, computational simulation methods are applied [8, 9]. Eleven factors have been experimentally reported to be related to cardiac alternans [8]. In order to find out the most relevant factors, investigators compared the differences of these factors between normal and alternans groups [8]. There are significant differences in the following 6 ionic currents between the two groups: the fast sodium current (*I*_Na_), the L-type calcium current (*I*_CaL_), the rapid delayed rectifier potassium current, the sodium calcium exchange current (*I*_NCX_), the sarcoplasmic reticulum (SR) calcium release current (*I*_rel_), and the SR calcium reuptake current (*I*_up_) [8, 9]. These 6 currents play an important role in the development of alternans. Voltage-driven alternans is related to APD restitution properties [1, 9]. APD restitution curve results from collective effects of the recovery properties of all the ionic currents and their interactions with membrane voltage [1, 5]. Sarcolemmal K^+^ and Ca^2+^ currents have an influence in the genesis of voltage-driven alternans. Transmembrane proteins such as Na^+^-Ca^2+^ exchange and Na^+^-K^+^ pump also take an effect [8]. Ca_*i*_ alternans is subsequently induced by the effect of voltage-dependent *I*_CaL_ current [6, 7]. Ca^2+^-driven alternans originates from steep fractional Ca^2+^ release relationship [10] or a generic mechanism of RyR properties, refractoriness, randomness, and recruitment [11]. *I*_rel_, *I*_up_, *I*_CaL_, and *I*_NCX_ have an effect on the genesis of Ca^2+^-driven alternans [6, 7]. A strong *I*_NCX_ can translate Ca_*i*_ alternans to voltage alternans [12].

Some of the 6 factors are related to instabilities of electrical activities in ischemia. Intracellular and extracellular acidosis affect ionic currents as channel proteins function like enzymes [13]. Conductibility of *I*_Na_ and *I*_CaL_ is decreased by acidosis. *I*_rel_ current is reduced significantly by acidosis [13]. SERCA2a is regulated by energy metabolism and its function is greatly decreased in ischemia [14]. A population-based study shows that the conductance *g*_CaL_ contributes most to the occurrence of APD alternans. Under ischemic conditions, there are also other currents such as *I*_NCX_, *I*_Kr_, and *I*_Ks_ that play a role [15].

Pathophysiological conditions in ischemia, such as hyperkalemia, acidosis, and hypoxia, promote alternans occurrence by affecting ionic currents at the cellular level. While many experimental and numerical studies reveal voltage- or Ca^2+^-dependent cellular mechanism, how ischemic conditions cause alternans remains unclear. In this work, we aim to investigate how electrical changes in ischemia promote alternans using computer simulations.

## 2. Methods

### 2.1. Hyperkalemic Condition

The epicardial ten Tusscher model (TNNP) [16] was employed in this study. In the epicardial cell model, we simulated hyperkalemic condition by increasing the extracellular potassium concentration ([K^+^]_o_). We changed [K^+^]_o_ alone to investigate its independent role in the development of alternans. [K^+^]_o_ concentration was set to increase from 5.4 to 15 mM. Cycle length was applied at 400 ms.

### 2.2. SERCA2a Function Decreased in Ischemia

A thermodynamic model of the cardiac SERCA2a [17] was integrated into the TNNP model. The thermodynamic model based on biophysical kinetic is sensitive to metabolism compromised in ischemia.

(1) 2Cai2++MgATP+H2O⟺2Casr2++MgADP+Pi+H+

The process of Ca uptake from the cytoplasm to SR can be presented by the above reaction equation. The equation shows that translating two Ca^2+^ needs the hydrolysis of one ATP. At the same time, the products MgADP, Pi, and H^+^ are released. This reversible reaction is modeled by E1-E2 model [18, 19] which consisted of two conformational changes of Ca^2+^-binding sites.

**Figure 1:**
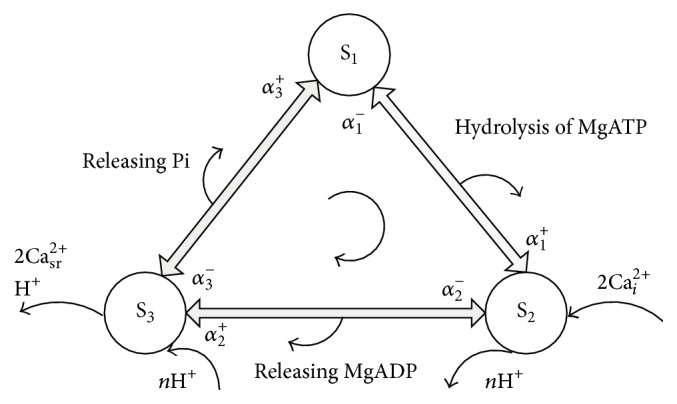
Schematic of the three-state model. S_1_, S_2_, and S_3_ represent the state of SERCA pump in the reaction process. The rate constants are represented by *α*_*i*_^±^(1,2, 3) and signs represent the forward or backward direction of reaction. Hydrolysis of MgATP and the release of MgADP/Pi occur in the positive direction during the transformation process. The reaction process is simplified from the E1-E2 model [18, 19].

The cardiac SERCA2a model applied in our study was a three-state model. The three-state model (Figure 1) is simplified from the E1-E2 model [18, 19] using rapid equilibrium assumption. In the positive direction, state S_1_ transforms to S_2_ via the hydrolysis of ATP. State S_2_ indicates Ca^2+^-binding sites binding Ca^2+^ and the Ca^2+^/H^+^ countertransport transporting H^+^ from SR to the cytoplasm, state S_3_ represents the Ca^2+^-binding sites releasing Ca^2+^ to the SR and Ca^2+^/H^+^ countertransport binding H^+^ to SERCA. The rate constants (*α*_*i*_) are functions of intracellular Pi, ATP, ADP, and H^+^ concentrations. See Appendix for formulas of these rate constants [17].

(2) $$\begin{array}{c} V_{cycle}=\frac{\alpha_{1}^{+}\alpha_{2}^{+}\alpha_{3}^{+}-\alpha_{1}^{-}\alpha_{2}^{-}\alpha_{3}^{-}}{\Sigma }, \end{array}$$

where *V*_cycle_ is clockwise cycle rate per pump at steady state. By modifying physiological parameters we could simulate metabolism compromised. In ischemia pH was decreased accompanied with a decrease of intracellular ATP. We set pH at 6 and ATP concentration at 4.2 mM. At the same time the concentrations of intracellular ADP and Pi [20] were simulated to increase to 100 nM and 30 mM [21], respectively. Values of other parameters in the formulas were the same as in the original three-state model [17].

(3) $$\begin{array}{c} I_{up}=N*V_{cycle}. \end{array}$$

The value of *I*_up_ was in proportion to the whole-cell pump flux. The whole-cell pump flux was determined by *V*_cycle_ and the numbers of SERCA pumps on the SR membrane. In order to calculate *I*_up_ under compromised metabolism conditions, we multiplied *V*_cycle_ by the constant *N* as a scale factor. The value of *N* was the ratio of maximum *I*_up_ obtained by original TNNP model simulation and maximum *V*_cycle_ under normal conditions.

## 3. Results

### 3.1. The Effects of Hyperkalemia on APD and Ionic Currents

**Figure 2:**
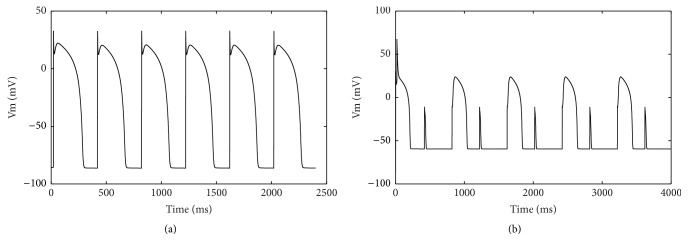
APD computed at the cycle length of 400 ms. (a) APs in control condition with [K^+^]_o_ = 5.4 mM. (b) Alternate APs in hyperkalemia with [K^+^]_o_ = 15 mM.

While the cycle length was applied at 400 ms, no APD alternans existed under normal conditions (Figure 2(a)). There existed no alternans except for the elevated resting potential and decreased amplitude of action potential when the [K^+^]_o_ values were in between 5 mM and 14.7 mM. APD alternans occurred in hyperkalemia with [K^+^]_o_ ranging from 14.7 to 15 mM. The [K^+^]_o_ values in this range correspond to severe hyperkalemia. Moreover, significantly elevated [K^+^]_o_ values may also occur in ischemic hearts as well as in isolated hearts in experiments. The longer AP manifested two depolarization phases. These two depolarization phases were maintained by *I*_Na_ and *I*_CaL_. The availability of *I*_CaL_ in shorter APs was reduced, resulting in small depolarization phases during the next beats (Figure 2(b)).

**Figure 3:**
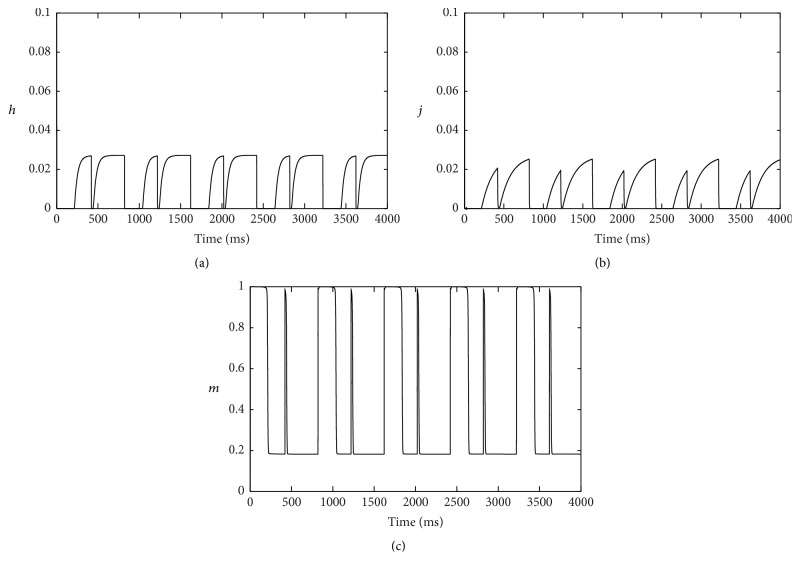
Gating variables of *I*_Na_ in hyperkalemia. (a) Fast inactivation gate *h*; (b) slow inactivation gate *j*; (c) activation gate *m*.

To investigate the process of alternans occurring, depolarization currents were selected to be studied. *I*_Na_ decreased significantly in hyperkalemia. Open possibilities of inactivation gates, *h* and *j*, came near to be zero (Figures 3(a) and 3(b)). In contrast, the open possibility of activation gate *m* increased at depolarized resting voltage (Figure 3(c)).

**Figure 4:**
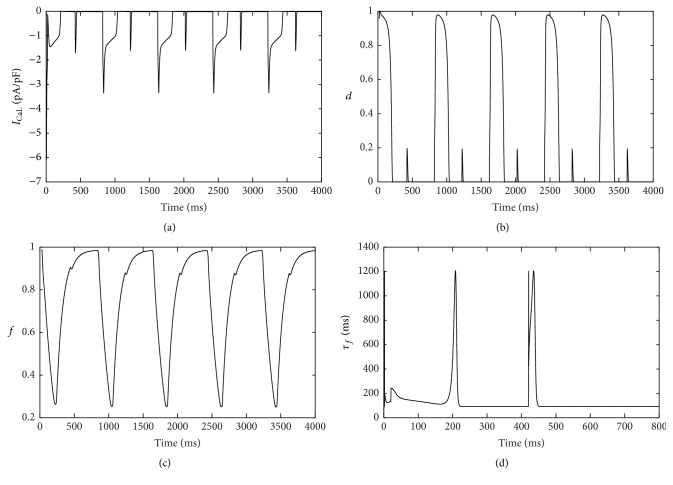
*I*_CaL_ and its gating variables in hyperkalemia. (a) *I*_CaL_ current; (b) voltage-dependent activation gate *d*; (c) voltage-dependent inactivation gate *f*; (d) inactivation time constant *τ*_*f*_ of the gate *f* during two consecutive beats.

**Figure 5:**
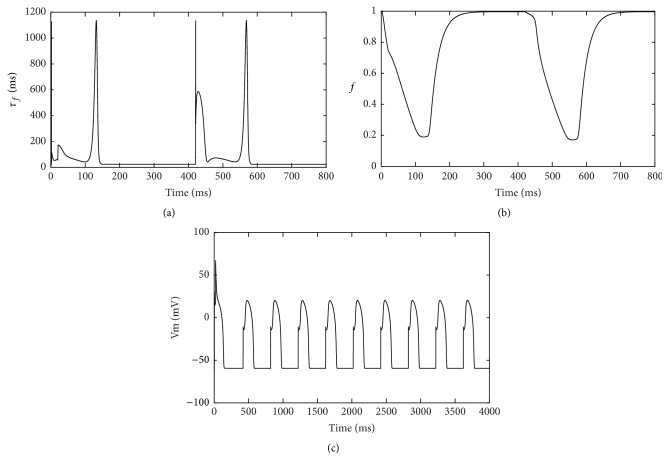
Simulations of the inactivation gate *f* and APD after decreasing inactivation time constant *τ*_*f*_ in hyperkalemia. (a) Decreased inactivation time constant *τ*_*f*_ of the gate *f*; (b) voltage-dependent inactivation gate *f*. Decreased *τ*_*f*_ made the gate *f* recover completely before the next APD. The gate *f* responded once during two consecutive beats in hyperkalemia without decreasing *τ*_*f*_; (c) APs with no alternans.

The amplitude of *I*_CaL_ showed alternans (Figure 4(a)). Activation gate *d* was voltage-dependent and manifested alternans from beat to beat (Figure 4(b)). The intracellular calcium-dependent inactivation gate, *f*_ca_, was nearly in closed state. Voltage-dependent inactivation gate *f* needed two cycle lengths to recover completely (Figure 4(c)). Moreover, the inactivation time constant *τ*_*f*_ of the gate *f* became larger during shorter APs (Figure 4(d)). That further verified the gate *f* could not recover instantly from inactivation, leading to decreased availability of *I*_CaL_ during shorter APs. While *τ*_*f*_ was decreased by 70 ms (Figure 5(a)), the gate *f* recovered instantly (Figure 5(b)) and alternans in APD disappeared (Figure 5(c)).

### 3.2. The Effects of *I*_up_ and *I*_NCX_ on Ca Transient and APD

**Figure 6:**
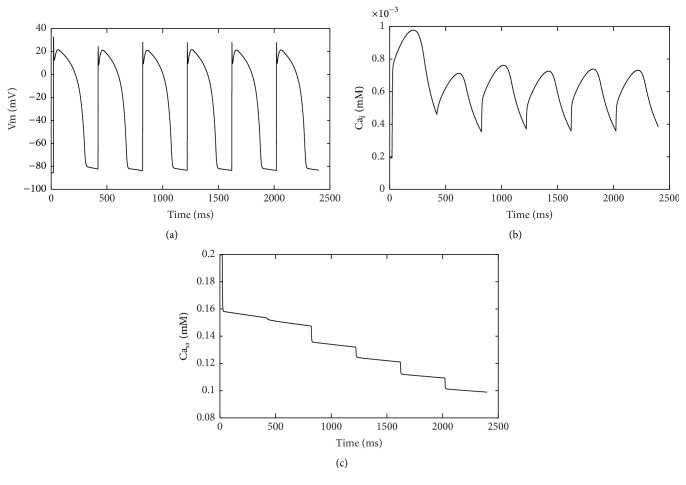
Ca transient, APs, and Ca^2+^ content in SR after decreasing *I*_up_ current. (a) APs with no alternans; (b) alternate Ca transient; (c) decreased Ca^2+^ content in SR from beat to beat.

As the component of cardiac Ca handling, *I*_up_ decreased under ischemic conditions. This change was simulated by modifying the physiological parameters of the SERCA pump model [17]. Thus the direct role of *I*_up_ in the onset of Ca_*i*_ alternans could be investigated. Decreased *I*_up_ slowed down the rate of SR Ca uptake and could not balance Ca^2+^ flux released from SR. As Figure 6(b) showed, Ca^2+^ transients alternated obviously during early beats and reached a steady state finally. In contrast to alternate Ca^2+^ transients, APD remained unchanged (Figure 6(a)).

**Figure 7:**
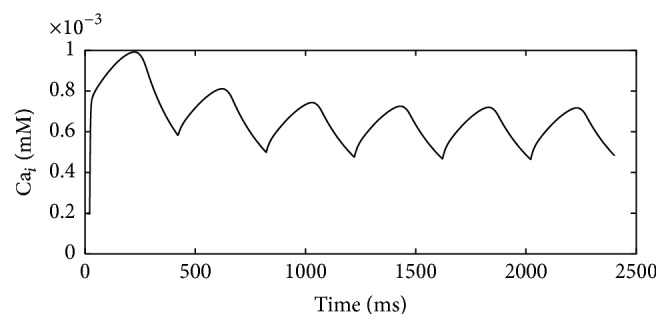
Ca transient after decreasing *I*_up_ and *I*_NCX_ currents. Ca_*i*_ alternans disappeared after decreasing *I*_NCX_.

**Figure 8:**
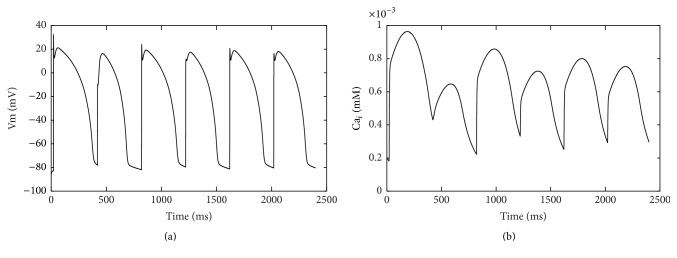
Ca transient and APs after decreasing *I*_up_ and increasing *I*_NCX_. (a) Alternate APs; (b) Ca_*i*_ alternans. Decreased *I*_up_ produced Ca_*i*_ alternans; however, the magnitude of Ca_*i*_ alternans was larger after increasing *I*_NCX_.

*I*_NCX_ decreased under acidic conditions [13]. Decreased *I*_NCX_ was also added in the simulation after investigating the effect of *I*_up_ on Ca_*i*_ alternans. As Figure 7 showed, the magnitude of Ca^2+^ transient alternans decreased. The result suggested that decreased *I*_NCX_ could inhibit Ca_*i*_ alternans. Based on this observation, we expected that Ca_*i*_ alternans magnitude would increase as *I*_NCX_ current increased.  Figure 8(b) confirmed the guess. Results showed that APD alternans was accompanied with Ca_*i*_ alternans of large magnitude (Figure 8(a)).

**Figure 9:**
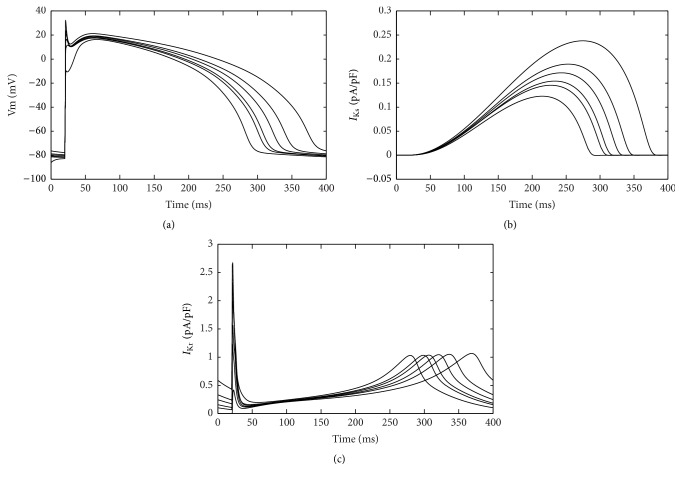
Alternate APs and two repolarization currents in consecutive beats after decreasing Iup and increasing *I*_NCX_. (a) Consecutive APs in the same coordinate system. Repolarization alternans was obvious by comparing duration of action potentials; (b) beat-to-beat alternation in repolarization current *I*_Ks_; (c) beat-to-beat alternation in repolarization current *I*_Kr_.

To compare the differences in the durations of repolarization between APs, we placed the 6 beats in the coordinate axes in Figure 9(a). Previous study suggested that *I*_Kr_ and *I*_Ks_ played a role in the occurrence of APD alternans. We selected *I*_Ks_ and *I*_Kr_ to investigate their roles in the process. *I*_Kr_ and *I*_Ks_ alternated from beat to beat as shown in Figures 9(b) and 9(c).

## 4. Discussion

### 4.1. The Mechanism of Alternans in Hyperkalemia

Depolarization alternans in hyperkalemia arises from changes in depolarization currents. In order to find out the key factors relating to alternans occurring in hyperkalemia, we selected depolarization currents for analysis. Our simulation results suggest that *I*_Na_ is too small to affect the process of depolarization during both longer and shorter APs. *I*_CaL_ may be the key factor in the development of alternans. Cycle lengths are fixed and the longer AP is followed by the shorter duration. *I*_CaL_ cannot recover completely from inactivation in the shorter duration. Its availability decreases in the following depolarization phase. Thus the next depolarization phase maintained by *I*_Na_ alone is small. Small depolarization phase leads to shorter AP. Subsequently, the longer duration provides enough time for *I*_CaL_ to recover completely. Shorter APs are following longer APs and alternans develops. In order to further verify the role of *I*_CaL_, *τ*_*f*_ of voltage-dependent inactivation gate *f* is decreased in simulation. Decreased *τ*_*f*_ indicates that the gate *f* needs shorter time to recover completely. Then the availability of *I*_CaL_ increases in APs. Alternans disappears due to complete response of *I*_CaL_ in every beat.

In contrast, some studies investigate alternans mechanisms in ischemia at the tissue level. Previous study supports that the depolarization alternans is linked to conduction abnormalities in the ischemia region [22]. The conduction block occurs under hyperkalemic conditions. Moreover, the depolarization phase is fragmented in the current simulation of hyperkalemia as is consistent with previous observations. Results show that depolarization alternans in ischemia region can be produced by hyperkalemic conditions [23].

Alternate conduction block induced by hyperkalemia leads to APD alternans [24]. The areas of conduction blocks become larger and alternans occurs at slower pacing frequency while increasing the inactivation time constant *τ*_*f*_ [24]. According to the observation, smaller areas are expected to be blocked if *τ*_*f*_ decreases and APD alternans will be depressed in the areas with no block any more. In other words, decreased *τ*_*f*_ can abolish alternans through eliminating conduction block. That is consistent with our observations. Hyperkalemia increases *τ*_*f*_ by depolarizing the resting voltage and thus promotes APD alternans.

### 4.2. The Direct Role of *I*_up_ in Ca_*i*_ Alternans

The slow rate of SR Ca uptake contributes to the occurrence of Ca_*i*_ alternans [25]. Qu et al. point out calcium alternans is determined by the interaction of the slopes of the fractional Ca^2+^ release curve, the SR Ca^2+^ uptake function, and properties of Ca^2+^ sparks [26]. The independent role of decreased *I*_up_ in the Ca_*i*_ alternans is investigated in our study. Decreased *I*_up_ has no ability to balance *I*_up_ current. The Ca content in diastole is affected. Subsequently, the release of Ca^2+^ from SR is depressed due to elevated Ca^2+^ in the cytoplasm. Then the Ca content in diastole decreases comparing to the last Ca transient. Fluctuations in cytoplasmic Ca content in diastole originate from unbalance in Ca^2+^ flux between *I*_up_ uptake and *I*_rel_ releasing. Transient Ca_*i*_ alternans are consequently caused by the fluctuations. According to the unified theory presented by Qu et al. [26], we could add ischemic changes of *I*_rel_ to the cell model to obtain stable calcium alternans. Ca content fluctuation in SR plays a role in producing Ca^2+^ transients alternans [10]. But our results show that SR load decreases from beat to beat (Figure 6(c)). That suggests SR load may not be the direct factor in the development of Ca_*i*_ alternans.

### 4.3. The Role of *I*_NCX_ in the Alternans Translation from Ca to APD

Larger *I*_NCX_ increases Ca_*i*_ alternans magnitude. Our results suggest that Ca_*i*_ alternans can lead to APD alternans while the Ca_*i*_ alternans magnitude is large enough. However, decreased *I*_up_ and increased *I*_NCX_ are not sufficient to produce stable alternans in our simulations. Previous study also shows that *I*_NCX_ is the key factor that translates alternans from Ca to APD [12]. More precisely, the balance of *I*_NCX_ and *I*_Ca_ determines coupling in phase of Ca_*i*_ alternans to APD alternans [27]. Alternans presented by Wan et al. can arise from the shifted balance of *I*_NCX_ and *I*_Ca_ at higher pacing rates. In our simulation, the extent of unbalance between these currents shifted by increasing *I*_NCX_ at cycle length of 400 ms could be too small to produce stable alternans [27]. *I*_Kr_ and *I*_Ks_ contribute to the occurrence of APD alternans in ischemia [15]. *I*_Kr_ contributes most due to its larger amplitude.

## 5. Conclusion

In silico simulations have been carried out to investigate cellular mechanisms of cardiac alternans under pathological disorders including hyperkalemia, acidosis, and hypoxia. Pathophysiological changes in ischemia play a significant role in the development of cardiac alternans by affecting ionic currents. Hyperkalemic conditions delay the recovery of depolarization current *I*_CaL_. Thus depolarization alternans occurs. Decreased *I*_up_ of Ca handling in ischemia promotes Ca_*i*_ alternans. A large *I*_NCX_ has the ability to translate alternans from Ca to APD. Studying changes of these ionic currents can help further understand cellular mechanisms of the genesis of alternans and form the basis of study of TWA in ischemia.

## Appendix

The rate constants used in (2) are as follows:

(A.1) α1+=k1+MgATP,α2+=k2+C~ai2C~ai2+1+H~i+H~i1+H~1,α3+=k3+H~srH~1+C~asr2+H~sr1+H~,α1−=k1−H~iC~ai2+1+H~i+H~i1+H~1,α2−=k2−MgADPC~asr2H~srH~1+C~asr2+H~sr1+H~,α3−=k3−Pi,

where

(A.2) C~ai=Ca2+iKd,Cai,H~i=H+Kd,Hi,H~1=H+Kd,H1,C~asr=Ca2+srKd,Casr,H~sr=H+Kd,Hsr,H~=H+Kd,H.
